# Prevalence of Substance Use Among Students of Senior Secondary Schools in the Tribal Sub-plan Area of Rajasthan

**DOI:** 10.7759/cureus.87815

**Published:** 2025-07-13

**Authors:** Chayani Pandya, Meenu Pichholiya, Arvind Yadav, Sakshi Singh, Mahendra Chaudhary

**Affiliations:** 1 Pharmacology, Geetanjali Medical College and Hospital, Udaipur, IND; 2 Pharmacology, American International Institute of Medical Sciences, Udaipur, IND

**Keywords:** adolescents, deaddiction, rajasthan, substance use, tribal sub-plan area

## Abstract

Introduction: Substance use among adolescents is a growing concern due to its impact on physical, mental, and cognitive health. Despite various studies on adolescent substance use in India, data regarding students in the tribal sub-plan (TSP) areas of Rajasthan remain limited, where the unique socio-economic and cultural factors influence these communities.

Objectives: To evaluate students’ knowledge and attitude regarding substance use and to assess the prevalence of substance use, along with the use of individual substances, among senior secondary school students in TSP areas.

Methods: A cross-sectional study was conducted after obtaining permission from the Institutional Ethics Committee of a tertiary care teaching hospital in Rajasthan, among students from classes nine to 12 over a six-month period (July-December 2024). Students of senior secondary schools in the TSP area in Rajasthan were included. A structured, validated questionnaire was used to assess the demographic profile of the participants and knowledge, attitudes, and practices of substance use among students. The data was collected through self-reported responses and analyzed using Microsoft Excel (Microsoft Corporation, Redmond, Washington, United States).

Results: A total of 260 students participated in this study, out of which 32 were substance abusers. The overall prevalence of substance use was 12.3% out of which tobacco users (239, 91.9%) and alcohol users (195, 75%) are the most common. Peer pressure was the primary reason for initiation, followed by tradition and curiosity. Out of all substance abusers, two (6.2%) experienced anxiety. Around 24 total substance abusers (75%) wanted to quit because they believed substances were harmful. However, 14 (43.7%) of them were willing to join de-addiction and rehabilitation programs to make positive changes in their lives.

Conclusion: The study concluded that substance abuse is a prevalent issue among adolescents, with tobacco emerging as the most commonly used substance. Many young individuals grappling with substance use disorders express a willingness to engage in de-addiction and rehabilitation programs, indicating a recognition of the need for help and a desire for change.

## Introduction

According to the World Health Organization (WHO), substance abuse refers to the harmful or hazardous use of psychoactive substances, including alcohol and illicit drugs [1]. The use of mood-altering psychoactive substances has been an integral aspect of human civilization for millennia. In India, substances such as alcohol, cannabis, and opioids have a long history of use, entwined with cultural practices and societal norms. This broad category of substance use encompasses not only the traditional consumption of these substances but also the misuse of prescription medications, as well as the use of tobacco, alcohol, and illicit drugs, including injections [2,3].

According to the United Nations Office on Drugs and Crime (UNODC) 2018 survey, drug use among young people is notably higher than among older individuals. Research indicates that adolescence, particularly between ages 12-17, is a critical-risk period for initiating substance use. Furthermore, substance use often peaks among individuals aged 18-25 years, highlighting the importance of targeted prevention strategies during these formative years to address and mitigate the risks associated with drug use. According to the Ministry of Social Justice and Empowerment’s 2019 report on the “National Survey on Extent and Pattern of Substance Use in India,” approximately 16 crore individuals (14.6%) aged 10 to 75 years were current alcohol users, with 5.2% classified as alcohol dependents. Additionally, around 3.1 crore individuals (2.8%) reported using cannabis, of which 72 lakh (0.66%) were identified as experiencing cannabis-related problems. These statistics highlight significant public health concerns regarding substance use and dependency in India [4].

Early substance use significantly affects the physical, mental, and cognitive development of adolescents, leading to behavioral issues and poor academic performance [5,6]. It disrupts critical developmental processes in the adolescent brain, particularly in areas responsible for decision-making and impulse control [7]. Research indicates that such early exposure can result in long-term detrimental effects on an individual’s well-being [8]. Therefore, recognizing and addressing early substance use through timely identification and intervention is crucial to mitigate these harmful consequences and promote healthier developmental trajectories for young people.

Much research has been done on the prevalence of substance use among adolescents in India [9]. But no such study had been conducted among students of senior secondary schools of tribal sub-plan (TSP) areas. TSP, now called the scheduled tribe component (STC), is a dedicated source of funds for tribal development, which includes support for education, health, livelihood, etc. [10]. The scheduled area in Rajasthan comprises eight districts of the southeastern part of Rajasthan, having a tribal population of more than 50 percent, as declared by the Government of India [11]. 

Recognizing the serious repercussions of substance use among children, alongside the insufficient data concerning substance use in senior secondary school students within TSP areas, this study was designed to evaluate students knowledge and attitude regarding substance use and to assess the prevalence of substance use, along with the use of individual substances, among senior secondary school students in TSP areas.

## Materials and methods

Study design

A cross-sectional study was conducted at Geetanjali Medical College and Hospital for six months (July-December 2024), after taking permission from the Human Research Ethics Committee of Geetanjali Medical College and Hospital (GU/HREC/EC/2024/244).

Inclusion criteria

Students enrolled in senior secondary schools located in the TSP area of Rajasthan. Students who were willing to provide informed consent for participation in the study.

Exclusion criteria

Students who were not willing to consent were excluded. Students who were absent on the day of data collection or unable to participate for any other reason.

Sample size

Sample size was estimated using the formula: $\frac{Z^2 p (1 - p)}{C^2}$ where Z stands for confidence level at 95%, which is equal to 1.96, p stands for prevalence, and C is the margin of error, i.e., 5%. After putting the values in the formula, the required minimum sample size was 210, considering a 20% nonresponse rate. 

Procedure

A questionnaire was prepared to assess the knowledge, attitude, and practices of substance use among students of senior secondary schools in TSP areas of Rajasthan with the help of other research questionnaires (Appendix 1) [12-14]. The questionnaire consists of three sections with items regarding the demographic profile of the participants and their knowledge, attitude, and practice of substance use. Multiple-choice questions were used throughout the questionnaire, allowing participants to easily provide their responses. This structured questionnaire was submitted to the medical education unit of our institute and experts in the field for validation. The suggestions from members of the medical education unit and experts in the field were considered, and the questionnaire was modified accordingly. The questionnaire was developed in English and also translated into the local language. Prior permission was taken from the head of the institution (school) for conducting the study. The validated questionnaire was given to students of senior secondary schools of the TSP area in Rajasthan. They were informed about the nature and purpose of the study, and assent/consent was taken beforehand. They were asked to fill out the questionnaire if they agreed to participate in the study, and return it blank if they were not willing to participate.

Statistical analysis

After gathering the data, it was entered and analyzed using Microsoft Excel (Microsoft Corporation, Redmond, Washington, United States). Data was presented in numbers or percentages of the responses provided. The chi-square test was used to analyze the qualitative data. A p-value less than 0.05 was considered to be significant.

## Results

The prevalence of substance abuse was 12.3% among the participants. A total of 260 students from classes nine to 12 participated, out of which 113 students (43.5%) were male, while 147 students (56.5%) were female. Around 117 (45%) students were from the age group 14-16 years. In the study, 161 students (61.9%) were from the 12th class, while seven students (2.69%) were from the 10th class. Of the students, 202 students (77.7%) lived with their parents. Almost all of the students, 256 (98.4%), were Hindus. Additionally, 205 (78.8%) belonged to the ST-TSP category. When it comes to education, 71 students (27.3%) had fathers who had completed secondary school, but 134 students (51.5%) had illiterate mothers. The sociodemographic details of the students showed that 162 students (62.3%) had fathers who worked as skilled laborers, while only a small number, 3 (1.1%), were retired. On the other hand, a vast majority of students' mothers, 243 (93.4%), were unemployed. Additionally, the household income for all participants was less than three lakhs. Table 1 shows the sociodemographic characteristics of the participants.

**Table 1:** Sociodemographic characteristics of all the study participants (N=260) ST-TSP: scheduled tribe-tribal sub-plan; SC: scheduled caste; OBC: other backward classes

Sr. No.	Sociodemographic characteristic	Number (%)
1	Gender	
	Male	113(43.5)
	Female	147(56.5)
2	Age group (years)	
	10-13years	21(8.1)
	14-16years	117(45)
	17-19years	122(46.9)
3	Grade	
	9^th^	44(16.9)
	10^th^	7(2.7)
	11^th^	48(18.5)
	12^th^	161(61.9)
4	Presently residing with	
	Parents	202(77.7)
	Guardian	8(3.1)
	In hostel	50(19.2)
5	Religion	
	Hindu	256(98.4)
	Muslim	2(0.8)
	Jain	2(0.8)
6	Marital status	
	Married	0(0)
	Unmarried	260(100)
7	Category	
	ST-TSP	205(78.8)
	SC	21(8.1)
	OBC	25(9.6)
	General	8(3.1)
	Minority	1(0.4)
8	Qualification of the father	
	Illiterate	83(31.9)
	Pre-primary	20(7.7)
	Primary	43(16.5)
	Secondary	71(27.3)
	Senior secondary	30(11.5)
	Graduate	13(5)
9	Qualification of the mother	
	Illiterate	134(51.5)
	Pre-primary	21(8.1)
	Primary	39(15)
	Secondary	38(14.6)
	Senior secondary	24(9.2)
	Graduate	4(1.5)
10	Father’s occupation	
	Professional	8(3.1)
	Skilled labourer	162(62.3)
	Semi-skilled labourer	2(0.8)
	Unskilled labourer	33(12.7)
	Unemployed	47(18.1)
	Retired	3(1.1)
	Deceased	5(1.9)
11	Mother's occupation	
	Professional	4(1.5)
	Skilled labourer	10(3.8)
	Semi-skilled labourer	1(0.4)
	Unskilled labourer	0(0)
	Unemployed	243(93.4)
	Retired	2(0.8)
12	Total household monthly income<3 lakhs	260(100)

All the participants were aware of substance abuse. Most of them, 239 (91.9%), were aware of tobacco, and only one of them (0.4%) knew about anabolic steroids and stimulants. Of them, 258 (99.2%) were aware of the harmful effects of substance use. Around 199 (76.5%) students were aware of cancer as a harmful effect of substance use. In the study, 102 (39.2%) of them gained the knowledge of adverse effects from TV, and 11 students (4.2%) from the internet. Among the students, 132 (50.8%) were unaware of the rehabilitation and de-addiction program. Analysis of questionnaire items based on knowledge in the study population is presented in Table 2. 

**Table 2:** Analysis of questionnaire items based on knowledge in the study population (N=260)

Sr. No.	Questionnaire items	Number (%)
1	Are you aware about substance use?	
	Yes	260(100)
	No	0(0)
2	Which of the following substances are you aware of?	
	Alcohol	195(75)
	Tobacco	239(91.9)
	Opioid	67(25.7)
	Anabolic steroid	1(0.4)
	Stimulants	1(0.4)
3	Do you know about the harmful effects of the substances used by you?	
	Yes	258(99.2)
	No	2(0.8)
4	What are the harmful effects of substance use that you know about?	
	Cancer	199(76.5)
	No response	61(23.4)
5	What is the source of knowledge about its harmful effects?	
	TV	102(39.2)
	Internet	11(4.2)
	Friends	43(16.5)
	Teachers	32(12.3)
	Family	11(4.2)
6	Are you aware of the rehabilitation and de-addiction programme?	
	Yes	128(49.2)
	No	132(50.8)

Out of all students, 32 were substance abusers. Of them, 15 (46.9%) started to try substances between the ages of 14 and 16 years. Around 20 (62.5%) have used tobacco, while a smaller group of two (6.25%) substance abusers have experimented with opioids. Seventeen (53.1%) of them have been using substances for less than a year, while six (18.7%) of them have been using them for more than five years. Additionally, half of the students used substances occasionally, and eight (25%) of them used them on a weekly basis. All abusers accessed substances from nearby shops, with 23 (71.9%) of them borrowing money from friends and relatives. A large majority, 31 (96.9%) abusers, have never been in debt. Some students, 20 (62.5%), reported feeling euphoria, and 10 (31.2%) pleasure from using substances. Peer pressure influenced 20 (62.5%) of them to use these substances. While only two (6.2%) experienced anxiety, 24 (75%) wanted to quit because 11 (34.3%) believed substances were harmful. However, 43.7% were willing to join de-addiction and rehabilitation programs to make positive changes in their lives. Analysis of questionnaire items based on attitude and practice in substance users is detailed in Table 3.

**Table 3:** Analysis of questionnaire items based on attitude and practice in substance users (n=32)

Sr. No.	Questionnaire item	Total n=32 (%)	P-value
1	What was your age at first attempt (10-18years)?		
	10-13years	13(40.6)	<0.05
	14-16years	15(46.9)	
	17-19years	4(12.5)	
2	Which substance have you ever used?		
	Alcohol	10(31.2)	<0.05
	Tobacco	20(62.5)	
	Opioid	2(6.25)	
3	How long have you been using substances?		
	<1year	17(53.1)	<0.05
	>1year	5(15.6)	
	>2year	4(12.5)	
	>5year	6(18.7)	
4	How frequently do you use a substance?		
	Just once	6(18.7)	<0.05
	Weekly	8(25)	
	Daily	2(6.25)	
	Monthly	0(0)	
	Occasionally	16(50)	
5	How do you access substance?		
	From nearby shops	32(100)	
6	How do you get money to buy substances?		
	Earned by fair means	5(15.6)	<0.05
	Borrowed from friends and relatives	23(71.9)	
	Pocket money	4(12.5)	
7	Have you ever been in debt?		
	Yes	1(3.1)	<0.05
	No	31(96.9)	
8	Which effects of substance use are experienced by you?		
	Euphoria	20(62.5)	<0.05
	Depression	1(3.1)	
	Disorientation	1(3.1)	
	Pleasure	10(31.2)	
9	Reason for using the substance use		
	Tradition	7(21.9)	<0.05
	Peer pressure	20(62.5)	
	Curiosity	5(15.6)	
10	Do you experience any of these symptoms upon the abrupt stoppage of substance use?		
	Increased heartbeat	1(3.1)	>0.05
	Anxiety	2(6.2)	
11	Do you want to quit substances?		
	Yes	24(75)	<0.05
	No	8(25)	
12	If yes, why do you want to quit substances?		
	Health issues	2(6.25)	<0.05
	Its harmful	11(34.3)	
13	Are you ready for de-addiction and rehabilitation programme?		
	Yes	14(43.7)	>0.05
	No	18(56.2)	

## Discussion

Substance abuse has emerged as a prevalent issue among students, particularly in underserved areas such as those within the TSP. Despite its significance, data on the extent and impact of substance abuse in these regions remain scarce, highlighting a critical gap in research that needs to be addressed. The current study reported a 12.3% prevalence of substance use among 10-19-year-olds in the TSP area of Rajasthan, comparable to Tsering et al. [12]. Higher prevalence of substance use was observed in studies by Juyal et al. (58.7%) and Sarangi et al. (43.4%), which could be potentially due to the regional acceptance of tobacco [15,16].

In the present study, substance use was notably more prevalent among males, which was similar to studies conducted by Kapoor et al., Madu et al., and Chen et al. [17,18,19]. This can be attributed to the social acceptance of substance use among males, which often influences behaviors and attitudes. Males may face fewer societal stigmas when engaging in these activities, leading to higher rates of use compared to females. This study highlighted a concerning trend in substance abuse among students aged 14 to 16 years, suggesting that peer influence during the teenage years plays a significant role in this behavior. Peer pressure has emerged as one of the most significant factors influencing substance use among individuals in this study, aligning with findings from Hembram et al. [20].

Many teenagers in this age group are reportedly encouraged by friends to experiment with drugs or alcohol, leading to increased incidents of substance abuse. This observation presents a contrast to previous research conducted by Saxena et al. and Rehman et al., which found that 45.1% and 48.15% of substance abusers were in the age category of 16 to 18 years [13,21]. This disparity raises important questions about the factors contributing to substance use among younger teens versus adolescents. Recognizing the difference in age groups is crucial for developing targeted prevention programs that address the specific needs and influences faced by students.

While a notable 91.9% of students were aware of tobacco use and 75% recognized the dangers of alcohol consumption, awareness regarding anabolic steroids and stimulants was significantly lower. Among these substances, tobacco emerged as the most commonly used, with 62.5% of substance abusers reporting its use. Students' easy access to tobacco products from nearby shops and their low cost contribute to this high prevalence. The affordability and availability of tobacco create an environment conducive to its higher usage compared to other substances. Supporting these findings, previous research by Mogan et al. and Saxena et al. has highlighted similar trends in substance awareness and usage among youth [7,13]. This indicates the need for targeted education and intervention programs to address not only tobacco use but also other substances, particularly anabolic steroids and stimulants, which may pose significant health risks to students. This study revealed that out of the total substance abusers, 53.1% of them initiated their substance use within the past year. Interestingly, despite the high percentage of new users, their substance consumption primarily tends to be occasional rather than frequent, similar to a study conducted by Anyanwu et al. [22]. This distinction suggests that while substance use is on the rise among recent initiates, it may not yet be entrenched into a daily habit, which could offer opportunities for early intervention. Understanding this pattern is crucial for developing effective prevention and treatment strategies. Educators, healthcare professionals, and policymakers can utilize this information to implement targeted programs aimed at reducing initiation rates and supporting at-risk individuals before occasional use escalates into more severe dependency issues.

It was found that a significant number (71.9%) of respondents relied on borrowing money from friends and family to meet their financial needs. Interestingly, despite this reliance on informal lending sources, only a small fraction of participants reported being in debt. This suggests that while people frequently turn to close connections for financial assistance, they may still be managing their overall financial health effectively, avoiding larger debts. This study revealed that an overwhelming 99.2% of participants recognized the harmful effects of the substances they used, with 76.5% specifically linking these effects to cancer. This finding aligns with previous research conducted by Kapoor et al. and Singh and Gupta, which indicated that substance abusers are generally aware of the risks posed by their habits [17,23]. The study also highlighted that a significant portion of respondents (39.2%) acquired their knowledge about these dangers primarily from television, which was similar to studies conducted by Mpabulungi et al. and Madan Kumar [24,25]. While only a small fraction (4.2%) obtained their information from the internet or family discussions. This suggests that the media plays a crucial role in educating individuals about the risks associated with substance use. The understanding of these risks among users is vital for promoting healthier choices and increasing awareness about the serious consequences that can arise from substance abuse. Efforts to enhance education on this topic could further empower individuals to make informed decisions regarding their health.

This study has found that a significant majority of participants (75%) communicated a strong willingness to quit substance use, highlighting their desire to change their lives for the better. This overwhelming response indicates a growing awareness among individuals regarding the harmful effects of substance use on their health and overall well-being. This finding aligns with previous research conducted by Mpabulungi et al., which found that 76.9% of smokers were also willing to quit, suggesting a strong motivation among smokers to improve their health [24]. However, contrasting results were reported by Franco et al., who discovered that only 33.5% of smokers showed a desire to quit, highlighting a potential disparity in willingness to change behaviors among different populations [26].

It was found that more than half of the students were not aware of rehabilitation and de-addiction programs available to them. This lack of awareness is primarily attributed to the insufficient number of programs conducted on substance abuse education and support. The study revealed that 56.2% of substance abusers expressed that they were not ready to engage in de-addiction and rehabilitation initiatives, indicating a significant need for increased outreach and education. This was in contrast to a study conducted by Singh and Gupta, where most of them were ready to participate [23]. Enhancing awareness through workshops, seminars, and informational campaigns could help break the stigma associated with seeking help. By fostering a supportive environment and providing accessible resources, it is possible to encourage those in need to consider participating in rehabilitation efforts, ultimately leading to healthier outcomes for individuals and the community as a whole.

In conclusion, this research concentrates on a specific demographic to illuminate the distinct challenges that these students encounter, particularly in relation to social, economic, and cultural factors that may heighten their risk of substance misuse. Through a thorough exploration of these underlying dynamics, the study aims to provide valuable insights that can inform the development of effective intervention strategies and support systems that cater to the unique needs of these communities. A nuanced understanding of the challenges faced by these students is crucial for implementing targeted awareness programs that address the root causes of substance use. By fostering an environment of support and education, it becomes possible to mitigate the impact of substance misuse, ultimately leading to healthier lifestyles and improved well-being. This focused approach not only benefits the individuals directly involved but also contributes positively to the broader community, promoting resilience and positive development among youth.

Limitations

The study encountered significant limitations due to its focus on a small sample size and a specific geographic area, particularly within a TSP region. This restricted scope may affect the generalizability of the findings, as the outcomes may not accurately reflect the experiences or conditions prevalent in broader populations or different settings. The small sample size can lead to amplified variability in the data and might not capture the full spectrum of factors influencing the subjects under study.

## Conclusions

The study concluded that substance abuse is a prevalent issue among adolescents, with tobacco emerging as the most commonly used substance. Many young individuals grappling with substance use disorders express a willingness to engage in de-addiction and rehabilitation programs, indicating a recognition of the need for help and a desire for change. These programs offer a crucial framework for support, education, and recovery, tailored to address the specific needs of adolescents. Early intervention is vital, as it can help mitigate the long-term effects of substance abuse on physical and mental health. As we move forward, collaboration among educators, parents, and healthcare professionals will be critical in shaping a brighter future for our youth.

## Appendices

Appendix 1 

**Table 4:** Questionnaire items based on knowledge, attitudes, and practice

Sr. No.	Questionnaire items	Number (%)
Questionnaire items related to knowledge		
1	Are you aware of substance use?	
	Yes	
	No	
2	Which of the following substances are you aware of?	
	Alcohol	
	Tobacco	
	Opioid	
	Anabolic steroid	
	Stimulants	
3	Do you know about the harmful effects of the substances used by you?	
	Yes	
	No	
4	What are the harmful effects of substance use that you know about?	
	Cancer	
	No response	
5	What is the source of knowledge about its harmful effects?	
	TV	
	Internet	
	Friends	
	Teachers	
	Family	
6	Are you aware of the rehabilitation and de-addiction programme?	
	Yes	
	No	
Questionnaire items related to attitude and practice		
1	What was your age at first attempt(10-18years)?	
	10-13years	
	14-16years	
	17-19years	
2	Which substance have you ever used?	
	Alcohol	
	Tobacco	
	Opioid	
3	How long have you been using substances?	
	<1year	
	>1year	
	>2year	
	>5year	
4	How frequently do you use a substance?	
	Just once	
	Weekly	
	Daily	
	Monthly	
	Occasionally	
5	How do you access substance?	
	From nearby shops	
6	How do you get money to buy substances?	
	Earned by fair means	
	Borrowed from friends and relatives	
	Pocket money	
7	Have you ever been in debt?	
	Yes	
	No	
8	Which effects of substance use are experienced by you?	
	Euphoria	
	Depression	
	Disorientation	
	Pleasure	
9	Reason for using the substance use	
	Tradition	
	Peer pressure	
	Curiosity	
10	Do you experience any of these symptoms on the abrupt stoppage of substance use?	
	Increased heartbeat	
	anxiety	
11	Do you want to quit substances?	
	Yes	
	No	
12	If yes, why do you want to quit substances?	
	Health issues	
	Its harmful	
13	Are you ready for the de-addiction and rehabilitation programme?	
	Yes	
	No	
